# *DLG3* variants caused X-linked epilepsy with/without neurodevelopmental disorders and the genotype-phenotype correlation

**DOI:** 10.3389/fnmol.2023.1290919

**Published:** 2024-01-05

**Authors:** Yun-Yan He, Sheng Luo, Liang Jin, Peng-Yu Wang, Jie Xu, Hong-Liang Jiao, Hong-Jun Yan, Yao Wang, Qiong-Xiang Zhai, Jing-Jing Ji, Weng-Jun Zhang, Peng Zhou, Hua Li, Wei-Ping Liao, Song Lan, Lin Xu

**Affiliations:** ^1^Department of Neurology, Women and Children’s Hospital, Qingdao University, Qingdao, China; ^2^Department of Neurology, Institute of Neuroscience, Key Laboratory of Neurogenetics and Channelopathies of Guangdong Province and the Ministry of Education of China, The Second Affiliated Hospital, Guangzhou Medical University, Guangzhou, China; ^3^Department of Neurology, The Affiliated Nanhua Hospital, Hengyang Medical School, University of South China, Hengyang, China; ^4^Department of Neurosurgery, The First Affiliated Hospital of Zhengzhou University, Zhengzhou, China; ^5^Epilepsy Center, Guangdong 999 Brain Hospital, Guangzhou, China; ^6^Department of Pediatrics, Guangdong General Hospital, Guangdong Academy of Medical Sciences, Guangzhou, China; ^7^Department of Neurology, Maoming People’s Hospital, Maoming, China

**Keywords:** *DLG3* gene, epilepsy, neurodevelopmental disorder, variants, Genotype-phenotype correlation

## Abstract

**Background:**

The *DLG3* gene encodes disks large membrane-associated guanylate kinase scaffold protein 3, which plays essential roles in the clustering of N-methyl-D-aspartate receptors (NMDARs) at excitatory synapses. Previously, *DLG3* has been identified as the causative gene of X-linked intellectual developmental disorder—90 (XLID-90; OMIM# 300850). This study aims to explore the phenotypic spectrum of *DLG3* and the genotype-phenotype correlation.

**Methods:**

Trios-based whole-exome sequencing was performed in patients with epilepsy of unknown causes. To analyze the genotype-phenotype correlations, previously reported *DLG3* variants were systematically reviewed.

**Results:**

*DLG3* variants were identified in seven unrelated cases with epilepsy. These variants had no hemizygous frequencies in controls. All variants were predicted to be damaging by silico tools and alter the hydrogen bonds with surrounding residues and/or protein stability. Four cases mainly presented with generalized seizures, including generalized tonic-clonic and myoclonic seizures, and the other three cases exhibited secondary generalized tonic-clonic seizures and focal seizures. Multifocal discharges were recorded in all cases during electroencephalography monitoring, including the four cases with generalized discharges initially but multifocal discharges after drug treating. Protein-protein interaction network analysis revealed that *DLG3* interacts with 52 genes with high confidence, in which the majority of disease-causing genes were associated with a wide spectrum of neurodevelopmental disorder (NDD) and epilepsy. Three patients with variants locating outside functional domains all achieved seizure-free, while the four patients with variants locating in functional domains presented poor control of seizures. Analysis of previously reported cases revealed that patients with non-null variants presented higher percentages of epilepsy than those with null variants, suggesting a genotype-phenotype correlation.

**Significance:**

This study suggested that *DLG3* variants were associated with epilepsy with/without NDD, expanding the phenotypic spectrum of *DLG3*. The observed genotype-phenotype correlation potentially contributes to the understanding of the underlying mechanisms driving phenotypic variation.

## 1 Introduction

Neurodevelopmental disorder (NDD) is the most common neurological disease in children, including intellectual development disorder (ID), developmental speech or language disorder (DSD), autism spectrum disorder (ASD), developmental learning disorder (DLD), attention deficit hyperactivity disorder (ADHD), tic disorder (TD) and others (Zhu et al., 2023). Epilepsy is one of the most common comorbidities in patients with neurodevelopmental disorders, whereas up to 26% of individuals with NDDs have epilepsy (McGrother et al., 2006; Ali Rodriguez et al., 2018; Heyne et al., 2018; Turner et al., 2021). NDD is a vital risk factor for epileptogenesis, and frequent seizures can also lead to or worsen NDD (Pisella et al., 2019). Increasing evidence has highlighted the genetic overlap of both epilepsy and NDD (Shimizu et al., 2022). Previously, a series of genes have been identified as causative genes for both epilepsy and NDD, such as *GRIN2A*, *GRIN2B*, *BCOR*, *FRMPD4*, *APC2*, *NEXMIF*, *SZT2*, *SHROOM4*, *BRWD3*, *KCNK4*, and *UNC79* (Liu et al., 2021; Bian et al., 2022; Li et al., 2022, 2023; Yan et al., 2022; Bayat et al., 2023; Jin et al., 2023; Luo et al., 2023; Tian et al., 2023; Wang et al., 2023; Ye et al., 2023). However, the majority of overlapping genetic etiologies for epilepsy and NDD remain undetermined.

The N-methyl-D-aspartate receptor (NMDAR) is one of the main excitatory receptors in the central nervous system, with essential roles in regulating neuroplasticity, excitatory neurotransmission, and the development of learning and memory (Chen et al., 2021). Genes encoding NMDAR subunits (such as *GRIN1*, *GRIN2A*, and *GRIN2B*) have been identified to be associated with broad-spectrum phenotypes, including epilepsies, epilepsies with NDD, and NDD without seizures (Endele et al., 2010; Charron et al., 2022). Similar to NMDAR genes, an increasing number of genes encoding NMDAR-associated proteins have been identified to be associated with a broad phenotypic spectrum, such as *DLG4* and *SYNGAP1* (Moutton et al., 2018; Agarwal et al., 2019). The phenotypic spectrum of other genes encoding NMDAR-associated proteins warrants further study.

The *DLG3* gene (OMIM* 300189), located in Xq13.1, encodes disks large membrane-associated guanylate kinase scaffold protein 3. The *DLG3* protein, also known as synapse-associated protein 102 (SAP102), is an NMDAR-associated protein with essential roles in clustering of NMDARs at excitatory synapses and regulating cell proliferation. It is highly expressed in the human brain, particularly in the cortex.^1^ Hemizygous knockout of *DLG3* in mice led to abnormalities in the cortex and synapse morphology, impairment in spatial learning, and abnormal excitatory postsynaptic currents (Cuthbert et al., 2007). Previously, *DLG3* has been identified as the causative gene of X-linked intellectual developmental disorder—90 (XLID-90; OMIM# 300850). It is unknown whether *DLG3* is associated with epilepsy and shares a broad phenotypic spectrum similar to NMDAR/NMDAR-associated genes.

In this study, we performed trio-based whole-exome sequencing (WES) in a cohort of patients with childhood epilepsy without acquired causes. Six novel *DLG3* variants were identified in seven unrelated cases with heterogeneous epilepsies, including three with epilepsy and four with epilepsy and ID. Previously reported *DLG3* variants were systematically reviewed to explore the underlying mechanism of phenotypic heterogeneity. This study suggested that *DLG3* may be associated with epilepsy without neurodevelopmental disability.

## 2 Materials and methods

### 2.1 Patients

The patients were recruited from multiple centers through the platform of China Epilepsy Project 1.0, including the Women and Children’s Hospital affiliated with Qingdao University, the Second Affiliated Hospital of Guangzhou Medical University, the First Affiliated Hospital of Zhengzhou University, Maoming People’s Hospital, Guangdong 999 Brain Hospital, and Guangdong Province People’s Hospital. Patients with acquired epilepsy were excluded, such as trauma, immunity, and infection. Detailed clinical information, including disease progression, prognosis, personal history, family history and results from general and neurological examinations, was collected from patients or their families.

### 2.2 Whole-exon sequencing

Blood samples of the probands were collected to extract genomic DNA. Whole-exon sequencing was performed using a NextSeq500 sequencing instrument (Illumina, San Diego, CA, USA) following the standard procedures previously described (Wang et al., 2018). The sequencing data were generated using massively parallel sequencing with an average depth of > 125x and > 98% coverage of the capture region on the chip, ensuring the acquisition of high-quality reads. These reads were mapped to the Genome Reference Consortium Human genome build 37 by Burrows-Wheeler alignment. Variants were called and qualified with the Genome Analysis Toolkit (DePristo et al., 2011). Sanger sequences were used to validate candidate variants.

### 2.3 Genetic analysis

To identify potentially pathogenic variants, an individualized analytical approach was employed for each case, following the methodology outlined in our previous study (Li et al., 2021; Wang et al., 2021). We screened *DLG3* variants with the explainable origination for genetic diseases, including *de novo* and hemizygous mutations. All *DLG3* variants identified in this study were annotated into the reference transcript NM_021120.4.

### 2.4 Literature review and analysis of genotype-phenotype correlation

The *DLG3* variants and associated clinical information were systematically reviewed from the PubMed database and the Human Gene Mutation Database (HGMD) up to September 2023. Variants with undefined origination or unexplained origination for the occurrence of genetic diseases were excluded. Null variants that result in truncated protein were employed to identify variants, including canonical splice site variants frameshift, nonsense, and initiation codon (Richards et al., 2015). Other variants were classified into non-null variants, such as missense and intron variants.

### 2.5 Bioinformatic analyses

In order to evaluate the detrimental impact of candidate missense variants, protein modeling was conducted using the Iterative Threading ASSEmbly Refinement software (I-TASSER) (Yang and Zhang, 2015). PyMOL Molecular Graphics System (version 2.3.2; Schrödinger, LLC; New York, USA) was utilized to visualize and analyze the protein structure changes. The protein stability changes of each variant were predicted using the I-Mutant Suite server (Capriotti et al., 2005), which indicated the free energy change (ΔΔG). Negative ΔΔG values indicate a decrease in mutant protein stability. The VarSite web server was used to analyze amino acid and hydrophobicity changes (Laskowski et al., 2020).

### 2.6 Protein-protein interaction (PPI) network analysis

The protein-protein interaction (PPI) network of the *DLG3* protein was analyzed using the STRING database (version: 12.0; University of Zurich, Zurich, Switzerland) (Szklarczyk et al., 2023). The interactive genes with a confidence score ≥ 0.7 were taken into analysis. The PPI networks were visualized by Cytoscape (version 3.10.1).

### 2.7 Statistical analysis

Statistical analyses were conducting using R (version 4.0.3). The two-tailed Fisher’s exact test was used to compare the differences between groups. *P*-value < 0.05 was considered statistically significant.

## 3 Results

### 3.1 Identification of *DLG3* variants

**FIGURE 1:**
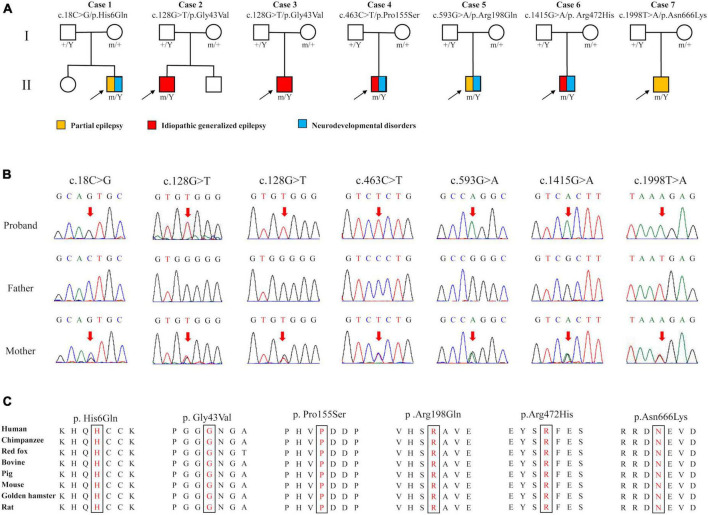
Genetic data of cases with *DLG3* variants. **(A)** Pedigrees of the cases with *DLG3* mutations and their corresponding phenotypes. **(B)** DNA sequencing chromatograms of the cases with *DLG3* variants. Red arrows indicate the positions of the mutations. **(C)** The amino acid sequence alignment showed that the six identified missense variants are residues with high conservation in mammals.

Six *DLG3* variants were identified in seven unrelated male individuals, including c.18C > G/p.His6Gln, c.128G > T/p.Gly43Val, c.463C > T/p.Pro155Ser, c.593G > A/p.Arg198Gln, c.1415G > A/p.Arg472His, and c.1998T > A/p.Asn666Lys (Figures 1A, B and Table 1). The variant c.128G > T/p.Gly43Val was recurrently identified in cases 2 and 3. All variants originated from their asymptomatic mothers, consistent with Mendelian X-linked recessive (XLR) inheritance.

**TABLE 1:** Clinical characteristics of individuals with *DLG3* variants.

Cases	Case 1	Case 2	Case 3	Case 4	Case 5	Case 6	Case 7
Variants (NM_021120.4)	p.His6Gln	p.Gly43Val	p.Gly43Val	p.Pro155Ser	p.Arg198Gln	p.Arg472His	p.Asn666Lys
Sex	M	M	M	M	M	M	M
Age	7 years	15 years	14 years	19 years	9 years	7 years	10 years
Seizure onset	2 years 2 month	4 years	12 years	16 years	7 years	4 years	2 years
Seizure course	sGTCS, 3–4 times/year	CPS, 2–3 times/day; GTCS and sGTCS, 3–4 times/month	GTCS and sGTCS, 1–2 times/year	GTCS, once; myoclonic seizure, 5–10 times/day	CPS, 5–6 times/month sGTCS, 3 times	GTCS 2 times/year	GTCS 2 times/month.
Prognosis	Seizure-free by VPA, LTG	Seizure-free by VPA, LTG	Seizure-free by LEV	Remission by VPA	Refractory	Seizure-free by VPA, LTG	Refractory
EEG	Diffuse slow waves in background; spike, poly-spike, and spike-slow waves in bilateral central-parietal-occipital regions and posterior temporal regions at 5 years old.	Generalized spike-slow waves at 4 years old. Slow wave paroxysm in the left frontal and frontal midline at 13-year-old. Normal at 15 years old.	Generalized spike and spike-slow waves at 12 years old. Multifocal spike and spike-slow waves at 13 years old. Normal at 14 years old.	Generalized sharp (spike) waves and poly-spike-slow waves.	Spike waves and spike-slow in bilateral anterior head at 7 years old.	Generalized spike and spike-slow waves at 4 years old. Multifocal spike and spike-slow waves at 7 years old.	Spike waves and spike-slow in left occipital and temporal lobe at 9 years old.
Brain MRI	NA	Normal	Normal	Gray matter heterotopia	Normal	NA	Normal
Neurodevelopment	ASD, DD and ID	Normal	Normal	Mild ID	ASD, ADHD, ID and speech delay	ID and speech delay	Normal
Diagnosis	PE	IGE	IGE	IGE	PE	IGE	PE

ADHD, attention deficit hyperactivity disorder; ASD, autism spectrum disorder; CPS, complex partial seizure; DD, developmental disorder; EEG, electroencephalogram; GTCS, generalized tonic-clonic seizure; ID, intellectual disability; IGE, idiopathic generalized epilepsy; PE, partial epilepsy; sGTCS, secondary generalized tonic-clonic seizure; LTG, lamotrigine; LEV, levetiracetam; MRI, magnetic resonance imaging; VPA, valproate acid.

These variants were absent in the male controls of the gnomAD database (Table 2). The six variants were found to be located in residues that were highly conserved among mammals based on amino acid sequence alignment (Figure 1C). All variants were predicted to be “damaging” or “conserved” by more than five *in silico* tools (Table 2).

**TABLE 2:** Genetic characteristics of *DLG3* mutations identified in this study.

Nucleotide change	Amino acid change	Inheritance	Hemizygous frequency*	PP2_ HDIV	PP2_ HVar	Mutation Taster	M_CAP	DANN	FATHMM_ MKL	GERP	phyloP	phastCons
c.18C > G	p.His6Gln	Maternal	0	PD (0.932)	PD (0.880)	DC (0.997)	D (0.305)	T (0.966)	D (0.916)	C (3.200)	C (4.967)	C (1.000)
c.128G > T	p.Gly43Val	Maternal	0	PD (0.999)	PD (0.972)	DC (0.994)	D (0.036)	T (0.979)	D (0.792)	NC (1.840)	C (2.475)	C (1.000)
c.463C > T	p.Pro155Ser	Maternal	0	PD (0.898)	PD (0.843)	DC (1.000)	D (0.048)	D (0.994)	D (0.958)	C (4.370)	C (2.444)	C (1.000)
c.593G > A	p.Arg198Gln	Maternal	0	PD (0.724)	B (0.311)	DC (0.971)	D (0.025)	D (0.996)	D (0.888)	C (4.480)	C (3.988)	C (1.000)
c.1415G > A	p.Arg472His	Maternal	0	PD (1.000)	PD (0.987)	DC (1.000)	D (0.119)	D (0.999)	D (0.881)	C (4.420)	C (9.602)	C (1.000)
c.1998T > A	p.Asn666Lys	Maternal	0	PD (0.974)	PD (0.828)	DC (1.000)	D (0.035)	T (0.982)	D (0.751)	NC (0.502)	NC (−0.102)	C (0.800)

*The number of hemizygous in the controls of gnomAD. B, benign; C, conserved; CADD, Combined Annotation Dependent Depletion; D, damaging; DANN, Domain-Adversarial Training of Neural Networks; DC, disease-causing; FATHMM-MKL, Functional Analysis through Hidden Markov Models–Multiple Kernels Learning; GERP, Genomic Evolutionary Rate Profiling; M_CAP, Mendelian Clinically Applicable Pathogenicity; NC, no-conserved; PD, probably damaging; phastCons, phylogenetic analysis with space/time models conservation scoring and identification of conserved elements; phyloP, phylogenetic analysis with space/time models computation of p-values for conservation or acceleration, either lineage-specific or across all branches; PP2_ HDIV, polyphen2_ HDIV; PP2_HVar, polyphen2_HVAR; T, tolerable.

**FIGURE 2:**
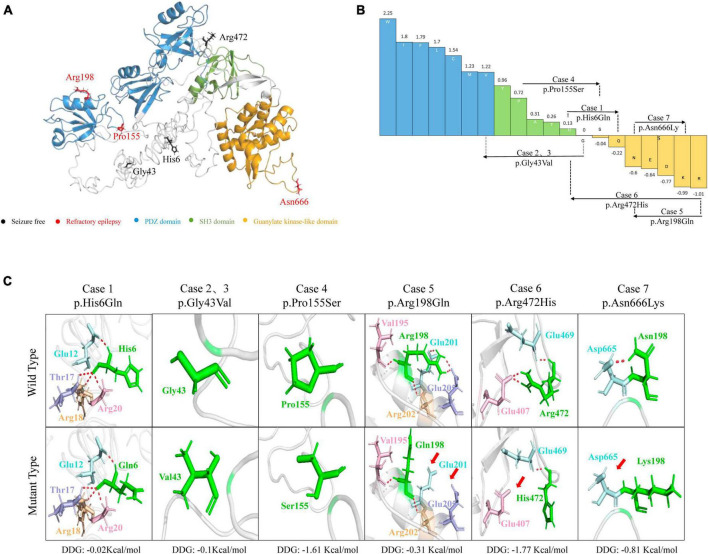
Schematic illustration of hydrogen bond changes, hydrophobicity changes and protein stability prediction. **(A)** Three-dimensional structure of *DLG3* protein and localization of *DLG3* variants of this study. **(B)** Fauchère and Pliska hydrophobicity scale exhibited the hydrophobicity of 20 amino acids. Abscissa: from left to right, hydrophobicity gradually decreased. Blue amino acids are hydrophobic, green amino acids are neutral, and yellow amino acids are hydrophilic. Amino acids with high positive values are more hydrophobic, whereas amino acids with low negative values are more hydrophilic. **(C)** Hydrogen bond changes and Gibbs free energy of folding (DDG) values of *DLG3* variants. The residues where the mutations occurred are shown as green rods. The red dotted line represents hydrogen bonds. Arrows indicate the positions with hydrogen bond changes. Three of six variants were predicted to alter hydrogen bonds with surrounding residues. All variants were predicted to decrease protein stability.

The molecular effects of the missense variants were assessed through protein modeling and visualized using PyMOL. Among the six missense variants, three were predicted to alter hydrogen bonds with neighboring residues. The remaining three missense variants (p.His6Gln, p.Gly43Val, and p.Pro155Ser) were not predicted to alter hydrogen bonds with surrounding residues but were predicted to decrease the protein stability (Figures 2A, C). Four variants (p.His6Gln, p.Gly43Val, p.Pro155Ser, and p.Arg472His) were also predicted to cause hydrophobicity changes, based on the Fauchère and Pliska hydrophobicity scale (Figure 2B).

No pathogenic or likely pathogenic variants in other epilepsy-related genes were identified in the seven patients (Supplementary Table 2) (Wang et al., 2017).

### 3.2 Clinical features of the cases with *DLG3* variants

**FIGURE 3:**
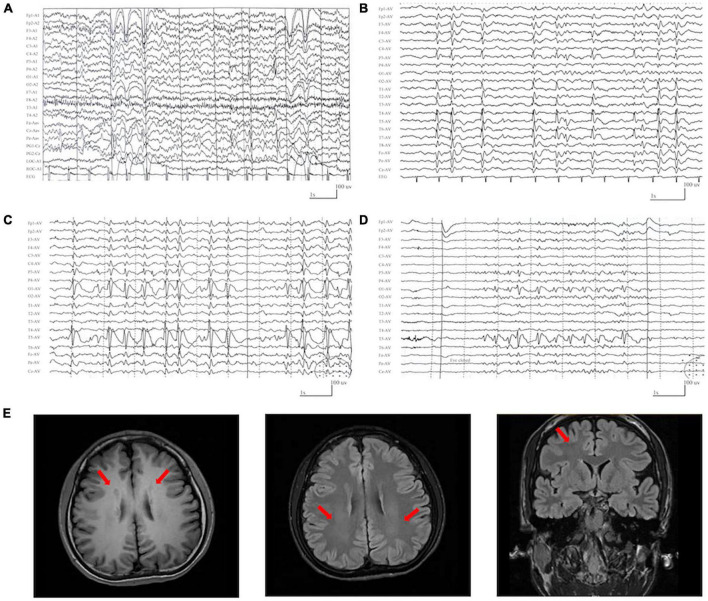
Representative EEG and magnetic resonance imaging (MRI) of the cases with *DLG3* variants. **(A)** Interictal EEG of case 1 at 2 years of age showed bilateral and multifocal spike-slow waves. **(B)** Interictal EEG of case 6 at 7 years of age showed bilateral multifocal spikes and spikes-slow waves (obtained). **(C,D)** Interictal EEG of case 7 at 9 years of age showed spike-slow waves predominant at the left posterior head **(C)** and eye closure sensitivity **(D)**. **(E)** The MRI of case 4 at the age of 16 years showed gray matter heterotopia.

The detailed clinical features of the patients with *DLG3* variants were summarized in Table 1. The patients were all diagnosed with epilepsy. The onset age of seizures ranged from 2 years old to 16 years old, with a median onset age of 4 years. Four cases mainly presented with generalized seizures (Cases 2, 3, 4 and 6), including generalized tonic-clonic and myoclonic seizures. Three cases (Cases 1, 5, and 7) exhibited secondary generalized tonic-clonic seizures and focal seizures. Seizure-free was achieved in cases 1, 2, 3, and 6. Multifocal discharges were recorded in all cases during EEG monitoring (Figures 3A–D). Generalized discharges were initially presented in cases 2, 3, 4 and 6, but multifocal discharges were also exhibited after being treated with antiseizure medications. MRI scans detected no structural abnormities of the brain, except case 4 with gray matter heterotopia (Figure 3E). Neurodevelopmental abnormalities were also exhibited in cases 1, 4, 5 and 6.

### 3.3 Location of *DLG3* variants and molecular subregion effects

**FIGURE 4:**
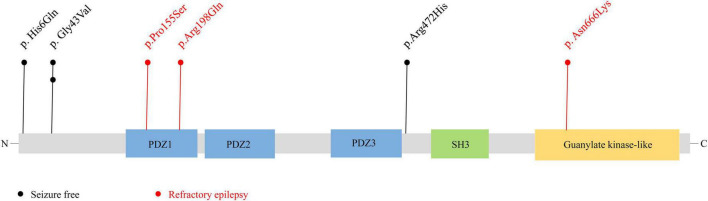
Schematic illustration of variant location. Schematic diagram of the *DLG3* protein and the localization of the *DLG3* variants identified in this study. Variants identified in patients with seizure-free are shown in black. Variants identified in patients with poor control of seizures are shown in red.

The SAP102 protein, contains three tandem amino-terminal PSD-95/Dlg/ZO-1 (PDZ) domains mediating protein-protein interactions, a src homology 3 (SH3) domain, and a C-terminal guanylate-kinase like (GK) domain (Kuwahara et al., 1999). The variants Pro155Ser and Arg198Gln were located in PDZ1 domain, and Asn666Lys was located in guanylate-kinase like domain, while other three variants (p.His6Gln, p.Gly43Val, and p.Arg472His) were located outside the functional domains (Figure 4).

Previous studies showed that the location of the variants was associated with the variation of clinical phenotypes (Tang et al., 2020). It is noted that the three variants locating outside functional domains were all identified in patients achieving seizure-free (Cases 1, 2, 3 and 6), while other three variants locating in functional domains were all identified in the patients with poor control of seizures (Case 4, 5 and 7).

### 3.4 Genotype-phenotype correlation

**FIGURE 5:**
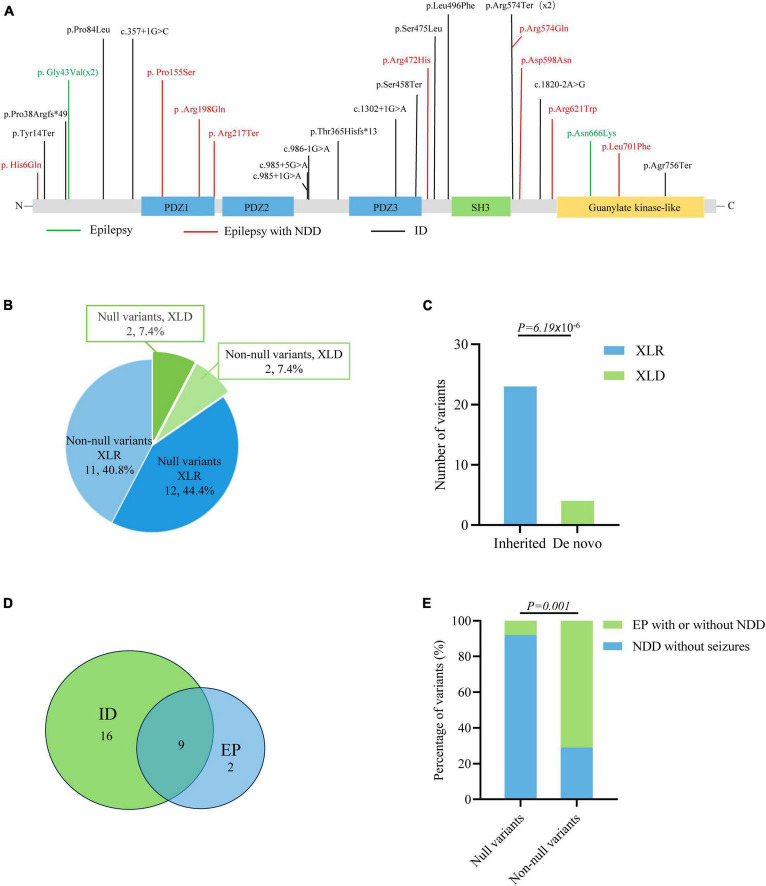
Genotype–phenotype correlation analysis of *DLG3* variants. **(A)** Schematic diagram of the *DLG3* protein and the localization of the *DLG3* variants identified in this study and reported previously. Variants associated with intellectual disability (ID) are shown in black, variants associated with epilepsy are shown in green, and variants associated with epilepsy and neurodevelopmental disorders (NDDs) are shown in red. * Means termination codon. **(B)** Pie chart of the genotype distribution of *DLG3* variants. **(C)** The stacked bar chart of inherited patterns and origination of the *DLG3* variants (*n* = 27). **(D)** Venn diagram of the phenotypes of patients with *DLG3* variants. A total of 27 variants were identified, including sixteen variants associated with intellectual disability, nine variants associated with both intellectual disability and epilepsy, and two variants associated with epilepsy. **(E)** The stacked bar chart of phenotypes of the variants with different genotypes. Variants with non-null variants presented higher percentages in patients with epilepsy with/without NDD than null variants (10/14 vs. 1/13; *p* = 0.001).

The previously reported variants and associated clinical information were systemically reviewed by using the HGMD (version: HGMD Professional 2023.1) and PubMed databases. A total of 27 variants were identified in 46 patients, including thirteen missense variants, five nonsense variants, five canonical splicing site variants, one intron variant, and three frameshift variants (Figure 5A and Supplementary Table 1). The majority of *DLG3* variants were of XLR pattern, and only four variants were of X-linked dominant (XLD) pattern. Further analysis showed that the XLR variants were all inherited from asymptomatic mothers, whereas XLD variants were all *de novo* (Figures 5B–D). The phenotype of male patients in the cases with XLD variants was more severe than that of female patients (Tarpey et al., 2004; Sandestig et al., 2020). To analyze the relationship between genotype and phenotype, the genotype was classified into null variants and non-null variants. Patients with non-null variants presented higher percentages of epilepsy with/without NDD than those with null variants (10/14 vs. 1/13; *p* = 0.001) (Figure 5E), potentially suggesting a genotype-phenotype correlation.

### 3.5 Protein-protein interactive network analysis

**FIGURE 6:**
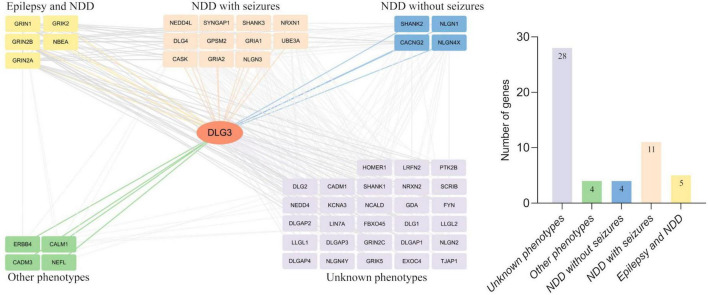
Analysis of genes interacting with *DLG3*. The *DLG3* protein interacted with 52 proteins with high confidence [**(left)**, minimum required interaction score ≥ 0.7, STRING database], including five genes identified to be associated with both epilepsy and neurodevelopmental disorder (NDD), eleven genes identified to be associated with NDD with seizures, four genes identified to be associated with NDD without seizures, four genes identified to be associated with other phenotypes, and 28 genes not identified to be associated with phenotypes **(right)**.

Protein-protein interactions, the basis of cellular metabolism, are indispensable in all life activities. The interactive genes tend to be associated with similar phenotypes. We thus further investigated the interactive partner of *DLG3* protein and their associated phenotypes to explore the underlying phenotypic spectrums of *DLG3* gene. *DLG3* protein is predicted to interact with 52 proteins with high confidence (minimum required interaction score ≥ 0.7, String database). The majority of disease-causing genes encoding the proteins interacting with the *DLG3* protein are associated with neurological diseases, including five causative genes of both epilepsy and NDD (*GRIN1*, *GRIN2A*, *GRIN2B*, *GRIK2*, and *NBEA*), eleven causative genes of NDD with seizures (*GRIA1*, *GRIA2*, *CASK*, *NRXN1*, *NLGN3*, *NEDD4L*, *SHANK3*, *GPSM2*, *DLG4*, *SYNGAP1*, and *UBE3A*), and four causative genes of NDD without seizures (*NLGN4X*, *CACNG2*, *SHANK2*, and *NLGN1*) (Figure 6). These broad phenotypes of genes encoding proteins interacting with *DLG3* protein provided possible clues for the association between *DLG3* and epilepsy.

## 4 Discussion

In this study, we identified six novel *DLG3* variants in seven unrelated patients with heterozygous epilepsies, including three with only epilepsy and four with epilepsy and NDD. The variants were not presented as hemizygous states in controls. These variants were located in residues with high conservation and were predicted to be damaging by multiple *in silico* tools. Protein modeling showed that the variants altered the hydron bonding with surrounding residues and protein stability. Genotype-phenotype correlation analysis revealed that patients with non-null variants presented higher percentages of epilepsy with/without NDD than those with null variants. This study suggested that *DLG3* variants were potentially associated with epilepsy with or without NDD. The genotype-phenotype correlation helps in understanding the underlying mechanism of phenotypic variation.

The *DLG3* gene was highly conserved with homologs in *Drosophila melanogaster*, *Mus musculus*, and *Homo sapiens*. In *Drosophila melanogaster*, the majority of knockout *DLG3* ortholog lines presented preadult lethality and abnormities in neuroanatomy neurophysiology (FlyBase ID: FBgn0001624). In *Mus musculus*, hemizygous knockout of *DLG3* led to abnormalities in the cortex and synapse morphology, impairment in spatial learning, and abnormal excitatory postsynaptic currents (Cuthbert et al., 2007). In *Homo sapiens*, data from large-scale genome sequencing reveal that *DLG3* is intolerant to loss-of-function (LOF) variants, with a probability of being LOF intolerant (pLi) of 1. The variants of the presented study were predicted to be “damaged” by diverse *in silico* tools and alter hydrogen bonding and/or polarity to disrupt protein stability, which may be associated with LOF. This evidence suggests that LOF may be the pathogenic mechanism for *DLG3*.

The *DLG3* protein is an important scaffold protein interacting with synaptic proteins, especially in excitatory synapses (Sheng and Hoogenraad, 2007; Murata and Constantine-Paton, 2013). Protein–protein interaction network analysis showed that *DLG3* interacted with 52 genes, of which 24 genes have been identified to be associated with disease. The sixteen genes are associated with NDD with seizures or developmental and epileptic encephalopathy (Figure 6). With increasing disease-causing variants identified, sixteen genes (including *GRIN1*, *GRIN2A*, *GRIN2B*, *GRIK2*, *NBEA*, *GRIA1*, *GRIA2*, *NEDD4L*, *CASK*, *NRXN1*, *NLGN3*, *SHANK3*, *GPSM2*, *DLG4*, *SYNGAP1*, and *UBE3A*) have been identified to be associated with a wide phenotypic spectrum from mild epilepsy to severe DEE/NDD with seizures (Endele et al., 2010; Harrison et al., 2011; Moog et al., 2011; Tan et al., 2011; Doherty et al., 2012; Soorya et al., 2013; Broix et al., 2016; Lemke et al., 2016; Moutton et al., 2018; Mulhern et al., 2018; Agarwal et al., 2019; Salpietro et al., 2019; Liu et al., 2021; Stolz et al., 2021; Blakes et al., 2022; Ismail et al., 2022). The wide phenotypic spectrum of *DLG3*-interacting genes provides possible clues for the association between *DLG3* and epilepsy. Previously, *DLG3* variants have been reported to be associated with XLID-90 (Supplementary Table 1). This study identified *DLG3* variants in three patients with mild epilepsy without NDD, broadening the phenotypic spectrum of *DLG3*.

The *DLG3* protein, contains three PDZ domains, a SH3 domain, and a GK domain. The PDZ domains interact with a diverse range of membrane proteins, such as ionotropic glutamate receptors, cell-surface adhesion molecules (Wei et al., 2018). The SH3 and GK domains interact with cytoskeletal proteins and intracellular signaling complexes (Wei et al., 2018). In this study, the three patients with variants locating outside functional domains all achieved seizure-free, while the four patients with variants locating in functional domains presented poor control of seizures, suggesting a molecular sub-regional effect. Compared with those in functional domain, variants locating out the functional domain may cause mild damaging effect and subsequently favorable outcomes, which may be one of the explanations of phenotypic severity.

**FIGURE 7:**
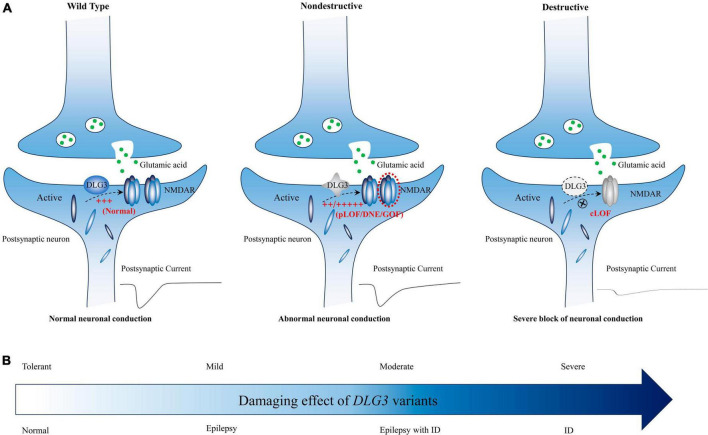
Schematic diagram of the possible association between *DLG3* variants on synaptic damage and related phenotypes. **(A)** Possible synaptic damage and postsynaptic current alteration of various *DLG3* variants. cLOF, complete loss of function; DNE, dominant-negative effects; GOF, gain of function; NMDARE, N-methyl-D-aspartate receptors; pLOF, partial loss of function. **(B)** The possible association between phenotypes and synaptic damage. Variants of mild or moderate damage would cause subtle functional alteration of synapse with abnormal electrophysiological activity and subsequently epilepsies and/or intellectual disability; while variants of complete loss of function would lead to decreased synaptic conduction, subsequently intellectual disability.

The *DLG3* protein, predominantly distributed in the postsynaptic densities of excitatory synapses, plays vital roles in synaptic development and synaptic transmission (Sans et al., 2000; Cuthbert et al., 2007; Elias and Nicoll, 2007; Elias et al., 2008; Zheng et al., 2011; Wei et al., 2015). Synaptic abnormalities are one of the core processes in the occurrence of neurodevelopmental disorders and epilepsies (Blanpied and Ehlers, 2004). The genotype-phenotype correlation analysis showed that patients with non-destructive variants mainly exhibited epilepsy with or without NDD, whereas patients with destructive variants primarily presented with intellectual disability (Figure 5). It is possible that phenotypic heterogeneity is associated with the damaging effect of *DLG3* variants (Figure 7). Variants of mild or moderate damage would cause subtle functional alteration of synapse with abnormal electrophysiological activity and subsequently epilepsies and/or intellectual disability; while variants of complete loss of function would lead to decreased synaptic conduction, subsequently intellectual disability. However, the functional alternations of all identified *DLG3* variants, including those previously reported variants, were not experimentally validated. The association between phenotypic heterogeneity and detailed mechanisms of variants, such as gain of function and dominant-negative effects, warrants functional studies.

The *DLG3* protein mediates NMDA receptor trafficking and contributes to NMDA receptor clustering and anchoring in the PSD (Lau et al., 1996; Nagano et al., 1998). Dysregulation of NMDAR subunits is one of the important mechanisms of partial epilepsy (Xu and Luo, 2018). In this study, the cases with *DLG3* variants all presented with multifocal discharges on EEG, in which four cases exhibited generalized discharges initially but also multifocal discharges after being treated with antiseizure medications. The phenotype of multifocal discharges potentially reflects the functional impact of *DLG3* variants in mediating NMDAR trafficking and may be one of the core features of patients with *DLG3* variants. However, the detailed functional alterations of these variants remain to be functionally validated.

In this study, gray matter heterotopia (GMH) was presented in the patient of case 4, which was distinguished from the other six cases. Abnormities of brain structure were also not presented in the previously reported cases (Supplementary Table 1). The *DLG3* variants may partially contribute to the phenotype of these cases but not gray matter heterotopia. However, Mendelian variants associated with cortical malformations were not identified in this case (Supplementary Table 2). It is unknown whether other factors are involved in the pathogenicity, which warrants further verification.

In summary, this study suggested that *DLG3* variants were associated with epilepsy with/without NDD, expanding the phenotypic spectrum of *DLG3*. The observed genotype-phenotype correlation contributes to our understanding of the underlying mechanisms of phenotypic variation.

## Data availability statement

The data presented in the study are deposited in the NCBI-GenBank database, accession numbers OR818645 and OR818665.

## Ethics statement

The studies involving human participants were reviewed and approved by the Ethics Committee of the Women and Children’s Hospital Affiliated with Qingdao University. Written informed consent to participate in this study was provided by the participant’s legal guardian/next of kin. The studies were conducted in accordance with the local legislation and institutional requirements. Written informed consent for participation in this study was provided by the participants’ legal guardians/next of kin. Written informed consent was obtained from the individual(s), and minor(s)’ legal guardian/next of kin, for the publication of any potentially identifiable images or data included in this article.

## Author contributions

Y-YH: Conceptualization, Data curation, Formal analysis, Investigation, Methodology, Project administration, Resources, Software, Supervision, Validation, Visualization, Writing – original draft, Writing – review & editing. ShL: Conceptualization, Data curation, Formal analysis, Investigation, Methodology, Project administration, Resources, Software, Supervision, Validation, Visualization, Writing – original draft, Writing – review & editing. LJ: Software, Writing – review & editing. P-YW: Methodology, Writing – review & editing. JX: Resources, Writing – review & editing. H-LJ: Resources, Writing – review & editing. H-JY: Resources, Writing – review & editing. YW: Resources, Writing – review & editing. Q-XZ: Resources, Writing – review & editing. J-JJ: Resources, Writing – review & editing. W-JZ: Resources, Writing – review & editing. PZ: Resources, Writing – review & editing. HL: Resources, Writing – review & editing. W-PL: Writing – review & editing. SoL: Resources, Writing – review & editing. LX: Writing – review & editing, Funding acquisition, Methodology, Resources.

## Footnotes

1
http://www.gtexportal.org/

## Web resources

OMIM: https://omim.org/.
GTEx database: https://www.gtexportal.org/.
PubMed database: http://www.ncbi.nlm.nih.gov/pubmed/.
Human Gene Mutation Database (HGMD): http://www.hgmd.cf.ac.uk/ac/index.php.
Iterative Threading ASSEmbly Refinement (I-TASSER): https://zhanglab.ccmb.med.umich.edu/I-TASSER/.
VarSite database: VarSite home page (ebi.ac.uk).
I-Mutant Suite server: https://folding.biofold.org/i-mutant/i-mutant2.0.html.
STRING database: https://string-db.org/.
FlyBase database: http://flybase.org/.
Mouse Genome Informatics (MGI): http://www.informatics.jax.org/.
Genome Aggregation Database (GnomAD): https://gnomad.broadinstitute.org/.
