# Supplementary material for: DLG3 variants caused X-linked epilepsy with/without neurodevelopmental disorders and the genotype-phenotype correlation
Source: Front Mol Neurosci. 2024 Jan 5;16:1290919. doi: 10.3389/fnmol.2023.1290919 (PMC10796462; doi:10.3389/fnmol.2023.1290919)
Supplement: Supplementary file 1 [file Table_1.DOCX]

**Supplement table 1. Characteristics of previously reported cases with putative disease-causing *DLG3* variants.**

| Case number | Nucleotide | Protein | Gender | Minor allele frequency* | Inheritance | Reported phenotype | Reference |
| --- | --- | --- | --- | --- | --- | --- | --- |
| Case1 | c.251C>T | p.Pro84Leu | Male | 1.156x10^5 | Mather | Mental retardation, X-linked | [1] |
| Case2 | c.1424C>T | p.Ser475Leu | Male | 9.737x10^6 | Mather | Intellectual disability | [2] |
| Case3-II | c.1486C>T | p.Leu496Phe | Male | 0 | Mather | Intellectual disability | [3] |
| Case3-II | c.1486C>T | p.Leu496Phe | Male | 0 | Mather | Intellectual disability | [3] |
| Case4 | c.1721G>A | p.Arg574Gln | Male | 3.287x10^5 | Mather | Intellectual disability (moderate); seizures | [4] |
| Case5 | c.1792G>A | p.Asp598Asn | Male | - | Mather | Motor developmental delay, seizures or encephalopathy | [5] |
| Case6 | c.1861C>T | p.Arg621Trp | Female | - | ***De novo*** | Epileptic encephalopathy | [6] |
| Case7 | c.2101C>T | p.Leu701Phe | Female | - | ***De novo*** | Mental retardation, facial abnormalities, epilepsy | [7] |
| Case8 | c.42T>G | p.Tyr14Term | Male | - | Mather | Intellectual disability | [8] |
| Case9-I | c.649C>T | p. Arg217Term | Male | - | Mather | Intellectual disability, seizures | [9] |
| Case9-II | c.649C>T | p. Arg217Term | Male | - | Mather | Intellectual disability | [10] |
| Case10-I | c.1373C>G | p.Ser458Term | Male | - | Mather | Mental retardation | [11] |
| Case10-II | c.1373C>G | p.Ser458Term | Male | - | Mather | Mental retardation | [11] |
| Case11-I | c.1720C>T | p.Arg574Term | Male | - | Mather | Intellectual disability | [12] |
| Case11-II | c.1720C>T | p.Arg574Term | Female | - | ***De novo*** | Mild psychomotor development delay | [12] |
| Case12 | c.1720C>T | p.Arg574Term | Male | - | Mather | motor development, and moderate ID | [13] |
| Case13 | c.2266C>T | p.Arg756Term | Male | - | Mather | Intellectual disability | [14] |
| Case14-I | c.985+5G>A | 328 | Male | - | Mather | Mental retardation | [11] |
| Case14-II | c.985+5G>A | 328 | Male | - | Mather | Mental retardation | [11] |
| Case15-I | c.1302+1G>A | 434 | Male | - | Mather | Mental retardation | [11] |
| Case15-II | c.1302+1G>A | 434 | Male | - | Mather | Mental retardation | [11] |
| Case15-III | c.1302+1G>A | 434 | Female | - | ***De novo*** | Mental retardation | [11] |
| Case16-I | c.357+1G>C | 119 | Male | - | Mather | Intellectual disability, delayed motor and speech development | [15] |
| Case16-II | c.357+1G>C | 119 | Male | - | Mather | Intellectual disability, delayed motor and speech development | [15] |
| Case16-III | c.357+1G>C | 119 | Male | - | Mather | Intellectual disability | [15] |
| Case17-I | c.985+1G>A | 328 | Male | - | Mather | Intellectual disability, delayed speech development | [15] |
| Case17-II | c.985+1G>A | 328 | Male | - | Mather | Intellectual disability, delayed motor and speech development | [15] |
| Case17-III | c.985+1G>A | 328 | Male | - | Mather | Intellectual disability, delayed motor and speech development | [15] |
| Case18-I | c.986-1G>A |  | Male | - | Mather | Mental retardation | [16] |
| Case18-II | c.986-1G>A |  | Male | - | Mather | Mental retardation | [16] |
| Case19 | c.1820-2A>G |  | Male | - | Mather | Developmental delay and cognitive impairment | [17] |
| Case20 | c.113_117delCTTAC | p.Pro38Argfs*49 | Male | - | Mather | Intellectual disability, Neurodevelopmental disorder | [18] |
| Case21-I | c.1092dupC | p.Thr365Hisfs*13 | Male | - | Mather | Mental retardation | [11] |
| Case21-II | c.1092dupC | p.Thr365Hisfs*13 | Male | - | Mather | Mental retardation | [11] |
| Case21-III | c.1092dupC | p.Thr365Hisfs*13 | Male | - | Mather | Mental retardation | [11] |
| Case21-IV | c.1092dupC | p.Thr365Hisfs*13 | Male | - | Mather | Mental retardation | [11] |
| Case21-V | c.1092dupC | p.Thr365Hisfs*13 | Female | - | Mather | Mental retardation | [11] |
| Case22 | c.-8dupG |  | Male | - | Mather | Intellectual disability | [19] |

**References:**

[1] N.M. Marinakis, M. Svingou, D. Veltra, K. Kekou, C. Sofocleous, F.N. Tilemis, K. Kosma, E. Tsoutsou, H. Fryssira, and J. Traeger-Synodinos, Phenotype-driven variant filtration strategy in exome sequencing toward a high diagnostic yield and identification of 85 novel variants in 400 patients with rare Mendelian disorders. Am J Med Genet A 185 (2021) 2561-2571.

[2] N. Ibarluzea, A.B. Hoz, O. Villate, I. Llano, I. Ocio, I. Marti, M. Guitart, E. Gabau, F. Andrade, B. Gener, and M.I. Tejada, Targeted Next-Generation Sequencing in Patients with Suggestive X-Linked Intellectual Disability. Genes (Basel) 11 (2020).

[3] E.Z. Taskiran, B. Karaosmanoglu, C. Kosukcu, G. Urel-Demir, O. Akgun-Dogan, P.O. Simsek-Kiper, M. Alikasifoglu, K. Boduroglu, and G.E. Utine, Diagnostic yield of whole-exome sequencing in non-syndromic intellectual disability. J Intellect Disabil Res 65 (2021) 577-588.

[4] K.M. Bowling, M.L. Thompson, M.D. Amaral, C.R. Finnila, S.M. Hiatt, K.L. Engel, J.N. Cochran, K.B. Brothers, K.M. East, D.E. Gray, W.V. Kelley, N.E. Lamb, E.J. Lose, C.A. Rich, S. Simmons, J.S. Whittle, B.T. Weaver, A.S. Nesmith, R.M. Myers, G.S. Barsh, E.M. Bebin, and G.M. Cooper, Genomic diagnosis for children with intellectual disability and/or developmental delay. Genome Med 9 (2017) 43.

[5] A.T. van der Ven, J. Johannsen, F. Kortum, M. Wagner, K. Tsiakas, T. Bierhals, D. Lessel, T. Herget, K. Kloth, J. Lisfeld, T. Scholz, N. Obi, S. Wortmann, H. Prokisch, C. Kubisch, J. Denecke, R. Santer, and M. Hempel, Prevalence and clinical prediction of mitochondrial disorders in a large neuropediatric cohort. Clin Genet 100 (2021) 766-770.

[6] T. Jiang, J. Gao, L. Jiang, L. Xu, C. Zhao, X. Su, Y. Shen, W. Gu, X. Kong, Y. Yang, and F. Gao, Application of Trio-Whole Exome Sequencing in Genetic Diagnosis and Therapy in Chinese Children With Epilepsy. Front Mol Neurosci 14 (2021) 699574.

[7] Y. Chen, X. Tang, L. Liu, Q. Huang, L. Lin, G. Liu, and N. Xiao, Comprehensive genome sequencing analyses identify novel gene mutations and copy number variations associated with infant developmental delay or intellectual disability (DD/ID). Genes Dis 9 (2022) 1166-1169.

[8] H. Stranneheim, K. Lagerstedt-Robinson, M. Magnusson, M. Kvarnung, D. Nilsson, N. Lesko, M. Engvall, B.M. Anderlid, H. Arnell, C.B. Johansson, M. Barbaro, E. Bjorck, H. Bruhn, J. Eisfeldt, C. Freyer, G. Grigelioniene, P. Gustavsson, A. Hammarsjo, M. Hellstrom-Pigg, E. Iwarsson, A. Jemt, M. Laaksonen, S.L. Enoksson, H. Malmgren, K. Naess, M. Nordenskjold, M. Oscarson, M. Pettersson, C. Rasi, A. Rosenbaum, E. Sahlin, E. Sardh, T. Stodberg, B. Tesi, E. Tham, H. Thonberg, V. Tohonen, U. von Dobeln, D. Vassiliou, S. Vonlanthen, A.C. Wikstrom, J. Wincent, O. Winqvist, A. Wredenberg, S. Ygberg, R.H. Zetterstrom, P. Marits, M.J. Soller, A. Nordgren, V. Wirta, A. Lindstrand, and A. Wedell, Integration of whole genome sequencing into a healthcare setting: high diagnostic rates across multiple clinical entities in 3219 rare disease patients. Genome Med 13 (2021) 40.

[9] A. Tzschach, U. Grasshoff, S. Beck-Woedl, C. Dufke, C. Bauer, M. Kehrer, C. Evers, U. Moog, B. Oehl-Jaschkowitz, N. Di Donato, R. Maiwald, C. Jung, A. Kuechler, S. Schulz, P. Meinecke, S. Spranger, J. Kohlhase, J. Seidel, S. Reif, M. Rieger, A. Riess, M. Sturm, J. Bickmann, C. Schroeder, A. Dufke, O. Riess, and P. Bauer, Next-generation sequencing in X-linked intellectual disability. Eur J Hum Genet 23 (2015) 1513-8.

[10] A. Rauch, D. Wieczorek, E. Graf, T. Wieland, S. Endele, T. Schwarzmayr, B. Albrecht, D. Bartholdi, J. Beygo, N. Di Donato, A. Dufke, K. Cremer, M. Hempel, D. Horn, J. Hoyer, P. Joset, A. Ropke, U. Moog, A. Riess, C.T. Thiel, A. Tzschach, A. Wiesener, E. Wohlleber, C. Zweier, A.B. Ekici, A.M. Zink, A. Rump, C. Meisinger, H. Grallert, H. Sticht, A. Schenck, H. Engels, G. Rappold, E. Schrock, P. Wieacker, O. Riess, T. Meitinger, A. Reis, and T.M. Strom, Range of genetic mutations associated with severe non-syndromic sporadic intellectual disability: an exome sequencing study. Lancet 380 (2012) 1674-82.

[11] P. Tarpey, J. Parnau, M. Blow, H. Woffendin, G. Bignell, C. Cox, J. Cox, H. Davies, S. Edkins, S. Holden, A. Korny, U. Mallya, J. Moon, S. O'Meara, A. Parker, P. Stephens, C. Stevens, J. Teague, A. Donnelly, M. Mangelsdorf, J. Mulley, M. Partington, G. Turner, R. Stevenson, C. Schwartz, I. Young, D. Easton, M. Bobrow, P.A. Futreal, M.R. Stratton, J. Gecz, R. Wooster, and F.L. Raymond, Mutations in the DLG3 gene cause nonsyndromic X-linked mental retardation. Am J Hum Genet 75 (2004) 318-24.

[12] A. Sandestig, A. Green, J. Aronsson, K. Ellnebo, and M. Stefanova, A Novel DLG3 Mutation Expanding the Phenotype of X-Linked Intellectual Disability Caused by DLG3 Nonsense Variants. Mol Syndromol 10 (2020) 281-285.

[13] T. Froukh, O. Nafie, S.A.S. Al Hait, L. Laugwitz, J. Sommerfeld, M. Sturm, A. Baraghiti, T. Issa, A. Al-Nazer, P.A. Koch, J. Hanselmann, B. Kootz, P. Bauer, W. Al-Ameri, R. Abou Jamra, A.J. Alfrook, M. Hamadallah, L. Sofan, A. Riess, T.B. Haack, O. Riess, and R. Buchert, Genetic basis of neurodevelopmental disorders in 103 Jordanian families. Clin Genet 97 (2020) 621-627.

[14] L. Gieldon, L. Mackenroth, E. Betcheva-Krajcir, A. Rump, S. Beck-Wodl, J. Schallner, N. Di Donato, E. Schrock, and A. Tzschach, Skewed X-inactivation in a family with DLG3-associated X-linked intellectual disability. Am J Med Genet A 173 (2017) 2545-2550.

[15] A.K. Philips, A. Siren, K. Avela, M. Somer, M. Peippo, M. Ahvenainen, F. Doagu, M. Arvio, H. Kaariainen, H. Van Esch, G. Froyen, S.A. Haas, H. Hu, V.M. Kalscheuer, and I. Jarvela, X-exome sequencing in Finnish families with intellectual disability--four novel mutations and two novel syndromic phenotypes. Orphanet J Rare Dis 9 (2014) 49.

[16] G. Zanni, H. van Esch, A. Bensalem, Y. Saillour, K. Poirier, L. Castelnau, H.H. Ropers, A.P. de Brouwer, F. Laumonnier, J.P. Fryns, and J. Chelly, A novel mutation in the DLG3 gene encoding the synapse-associated protein 102 (SAP102) causes non-syndromic mental retardation. Neurogenetics 11 (2010) 251-5.

[17] X. Zhang, W. Qiu, H. Liu, X. Ye, Y. Sun, Y. Fan, and Y. Yu, RT-PCR analysis of mRNA revealed the splice-altering effect of rare intronic variants in monogenic disorders. Ann Hum Genet 84 (2020) 456-462.

[18] S.H. Kim, B. Kim, J.S. Lee, H.D. Kim, J.R. Choi, S.T. Lee, and H.C. Kang, Proband-Only Clinical Exome Sequencing for Neurodevelopmental Disabilities. Pediatr Neurol 99 (2019) 47-54.

[19] R. Kumar, T. Ha, D. Pham, M. Shaw, M. Mangelsdorf, K.L. Friend, L. Hobson, G. Turner, J. Boyle, M. Field, A. Hackett, M. Corbett, and J. Gecz, A non-coding variant in the 5' UTR of DLG3 attenuates protein translation to cause non-syndromic intellectual disability. Eur J Hum Genet 24 (2016) 1612-1616.
